# First-in-human phase 0 study of ^111^In-CHX-A”-DTPA trastuzumab for HER2 tumor imaging

**DOI:** 10.15761/JTS.1000269

**Published:** 2018-07-13

**Authors:** KA Kurdziel, E Mena, Y McKinney, K Wong, S Adler, T Sissung, J Lee, S Lipkowitz, L Lindenberg, B Turkbey, S Kummar, DE Milenic, JH Doroshow, WD Figg, MJ Merino, CH Paik, MW Brechbiel, PL Choyke

**Affiliations:** 1 Molecular Imaging Program (MIP), Center for Cancer Research (CCR)/National Cancer Institute (NCI), National Institutes of Health (NIH), USA; 2 Clinical Research Directorate/Clinical Monitoring Research Program, Leidos Biomedical Research, Inc., Frederick National Laboratory for Cancer Research, USA; 3 Genitourinary Malignancies Branch, CCR/NCI, NIH, USA; 4 Division of Nuclear Medicine, Radiology and Imaging Sciences, Clinical Center(CC), NIH, USA; 5 Women’s Malignancies Branch, CCR/NCI, NIH, USA; 6 Radiation Oncology Branch, CCR/NCI, NIH, USA; 7 Division of Cancer Treatment and Diagnosis and CCR/NCI, NIH, USA; 8 Laboratory of Pathology, CCR/NCI, NIH, USA

**Keywords:** molecular imaging, hercepti, trastuzumab, HER2, oncology, CHX-A”-DTPA

## Abstract

**Introduction:**

Tumors over-expressing the human epithelial receptor 2 (HER2) or exhibiting amplification or mutation of its proto-oncogene have a poorer prognosis. Using trastuzumab and/or other HER2 targeted therapies can increase overall survival in patients with HER2(+) tumors making it critical to accurately identify patients who may benefit. We report on a Phase 0 study of the imaging agent, ^111^In-CHX-A”-DTPA trastuzumab, in patients with known HER2 status to evaluate its safety and biodistribution and to obtain preliminary data regarding its ability to provide an accurate, whole-body, non-invasive means to determine HER2 status.

**Methods:**

^111^In-CHX-A”-DTPA trastuzumab was radiolabeled on-site and slowly infused into 11 patients who underwent single (n=5) or multiple (n=6) ɣ-camera (n=6) and/or SPECT (n=8) imaging sessions.

**Results:**

No safety issues were identified. Visual and semi-quantitative imaging data were concordant with tissue HER2 expression profiling in all but 1 patient. The biodistribution showed intense peak liver activity at the initial imaging timepoint (3.3h) and a single-phase clearance fit of the average time-activity curve (TAC) estimated t_1/2_=46.9h (R^2^=0.97; 95%CI 41.8 to 53h). This was followed by high gastrointestinal (GI) tract activity peaking by 52h. Linear regression predicted GI clearance by 201.2h (R^2^ =0.96; 95%CI 188.5 to 216.9h). Blood pool had lower activity with its maximum on the initial images. Non-linear regression fit projected a t_1/2_=34.2h (R^2^ =0.96; 95%CI 25.3 to 46.3h). Assuming linear whole-body clearance, linear regression projected complete elimination (x-intercept) at 256.5hr (R^2^=0.96; 95%CI 186.1 to 489.2h).

**Conclusion:**

^111^In-CHX-A”-DTPA trastuzumab can be safely imaged in humans. The biodistribution allowed for visual and semiquantitative analysis with results concordant with tissue expression profiling in 10 of 11 patients. Advances in Knowledge and Implications for Patient Care Using readily available components and on-site radiolabeling ^111^In-CHX-A”-DTPA trastuzumab SPECT imaging may provide an economical, non-invasive means to detect HER2 over-expression.

## Introduction

Amplification of the proto-oncogene, HER2/neu (aka: c-erb-B2) may increase the number of human epithelial receptor 2 (HER2) transmembrane protein receptors [1,2]. A member of the Epithelial Growth Factor Receptor (EGFR) subgroup (erbBl-4, a.k.a. EGFR/HER1, HER2, HER3 and HER4), HER2 is a 185kD transmembrane receptor tyrosine kinase (TK) [3] with a prominent extramembrane binding domain (EBD), a hydrophobic trans-membrane component and a smaller intracellular (cytoplasmic) component containing the TK domain. While it has no known natural ligands, HER2 can self-dimerize or heterodimerize with other EGFR members [4] enabling autophosphorylation and activation the TK domain initiating downstream intracellular signal transduction pathways that regulate cell proliferation, metabolism and cell survival [3]. While HER2 is required for normal cell function [5], its over-expression in breast and other tumors [6] is associated with decreased apoptosis [7], increased angiogenesis [8], progression and metastasis [9.^10^], recurrence [11] and overall poorer prognosis [12]. Trastuzumab, a 148kDa humanized monoclonal antibody selectively and specifically binds (k_d_=5nM) [13] to two extracellular juxta-membrane sites of HER2 inhibiting tumor proliferation. Binding also induces antibody-dependent cell-mediated cytotoxicity (ADCC) and antibody-dependent cell-mediated phagocytosis (ADCP) [14,15]. Trastuzumab is currently FDA-approved for use only in patients whose tumors overexpress HER2. In breast cancer, it is approved for use in the adjuvant or metastatic setting, as either a single agent or in specific combinations [16]. It is also approved for use in specified first-line combination therapies for patients with metastatic gastric or gastroesophageal junction adenocarcinoma [16].

Since trastuzumab’s approval, several other therapies targeting HER2 have emerged including: lapatinib (Tykerb), pertuzumab (Perjeta), ado-trastuzumab emtansine (Kadcyla) and neratinib (Nerlynx). With the growth in HER2 targeted therapies comes an increase in the desire to accurately, non-invasively determine the HER2 status of both primary and metastatic tumors. Currently, HER2 tumor expression level determination, positive/HER2(+) or negative/HER2(−), requires direct visualization of tumor tissue over-expressing HER2 on the cell membrane using immunohistochemistry (IHC) and/or identification of its proto-oncogene (erb-b2) amplification [2] using fluorescent in-situ hybridization (FISH) or similar method. Despite standardized testing methods and harmonized interpretation recommendations [17–20], diagnostic variability remains. Tissue samples are necessarily limited by the need for biopsy and sampling error leading to an underestimation bias of HER2 positivity due to intra- and inter-tumor heterogeneity [21].

We radiolabeled trastuzumab with a radioisotope of Indium (^111^In) that can be imaged by ɣ-camera planar whole-body or SPECT. ^111^In has an average physical half-life (t_1/2_) of 67.9h, therefore a 185MBq dose allows for imaging the uptake and clearance of trastuzumab throughout the entire imaging period (1-week) of the protocol. Prior reports of ^111^In-DTPA and ^111^In-MX-DTPA-trastuzumab have been published showing promising results [22–24]. Using the novel acyclic bifunctional chelate (BFC): 2-[[(1R)-2-[bis(carboxymethyl)amino]cyclohexyl]-[(2S)-2-[bis(carboxym ethyl) amino] −3-(4-isothiocyanatophenyl)propyl] amino]acetic acid, 594.6 g/mol, commonly referred to as CHX-A”-DPTA [25]. Its strong metal-chelating group and chemically reactive functional group enable it stably bind various metallic radionuclides (^111^In, ^90/86^Y, ^212/213^Bi,^177^Lu) to proteins allowing for both diagnostic and therapeutic applications. It has a higher serum stability [26], than its 2^nd^ generation predecessor Mx-DTPA (tiuxetan) which is used clinically in ^90^Y-ibritumomab (Zevlin) radioimmunotherapy for B-cell lymphoma [27]. There have been no safety issues in previous clinical trials using CHX-A”-DPTA [28–30] which is now commercialized, essentially replacing Mx-DTPA.

We present preliminary results of a Phase 0 ^111^In-CHX-A”-DTPA trastuzumab imaging study in cancer patients, including an evaluation of its safety, physiologic biodistribution over time, and comparison of lesion uptake with pathologic HER2 status.

## Materials and methods

### Patient population

Eligible participants were ≤18 years old, with history of primary or metastatic cancer (other than melanoma, basal cell carcinoma, sarcoma or lymphoma) and radiologically identifiable solid tumor ≥1.5cm with HER2 expression by IHC and/or FISH, or availability of a tumor specimen on which such an analysis could be performed. The study was approved by the local Investigational Review Board (IRB). Informed consent was obtained from all individual participants.

A total of 13 patients were enrolled: Median [minimum, maximum] age was 56 [35,68] years and weight 73 [56.9,76.8] kg. The imaged population was highly pre-treated for cancer and presented with locally recurrent and/or metastatic cancer: 8 patients had metastatic breast cancer, 2 patients had metastatic non-small cell lung cancer (NSCLC) and 1 patient had metastatic bladder cancer. The population included 3 HER2(−) and 8 HER2(+) patients. Eight patients had prior trastuzumab exposure and 3 were receiving trastuzumab therapy throughout this study. The patients were not pretreated with non-radiolabeled antibody. ^111^In-CHX-A”-DTPA trastuzumab was administered IV over 10-15min followed by a normal saline flush. Continuous evaluation for potential adverse reactions and routine safety monitoring was performed prior to and during each imaging session. Evaluation for “shed” extracellular binding domain (EBD) in patient plasma was performed using the HER2 ELISA kit from Millipore Sigma (St. Louis, MO, USA). A blood sample obtained ~30-d post ^111^In-CHX-A”-DTPA-trastuzumab infusion was evaluated for immunogenicity (assess for potential antibodies to the experimental radiotracer) via HPLC (NCT01445054). Further details of the patient population are presented in (Table 1)

### Patient and tumor characteristics

Outside pathology reports were used to confirm malignancy and determine HER2 status for inclusion. As the final patient was enrolled in 2014, the 2013 American Society of Clinical Oncology (ASCO)/College of American Pathologists (CAP) recommendations for HER2 assessment in Breast Cancer were used [31]. Unstained slides or blocks of tissue were requested for repeat HER2 testing on-site. If not available, the existing slides were reviewed for accuracy. The on-site HER2 designation was considered “ground truth”.

### Radiochemistry

The non-radiolabeled precursor, BFC-antibody complex, CHX-A”-DTPA trastuzumab [NSC 741820] was synthesized using Good Manufacturing Practices (GMP) (Goodwin Biotechnology, Plantation, Florida) and provided by Cancer Therapeutics Evaluation Program, Division of Cancer Diagnosis and Treatment, National Cancer Institute. Radiolabeling with ^111^InCl (in dilute hydrogen chloride solution) was performed on site as previously published [32]. ^111^In -CHX-A”-DTPA trastuzumab [NSC 740377] was assessed for radiochemical purity, >95%; chemical mass, <200ug (<l/100^th^ of a clinical therapeutic dose) and radioactivity, ~185MBq. Small aliquots of each patient dose were used for counting, sterility assessment and Limulus Ameboctye Lysate (LAL) testing for endotoxin and other pathogen-associated molecular patterns (PAMPs).

### Imaging and analysis

**Figure 1.**
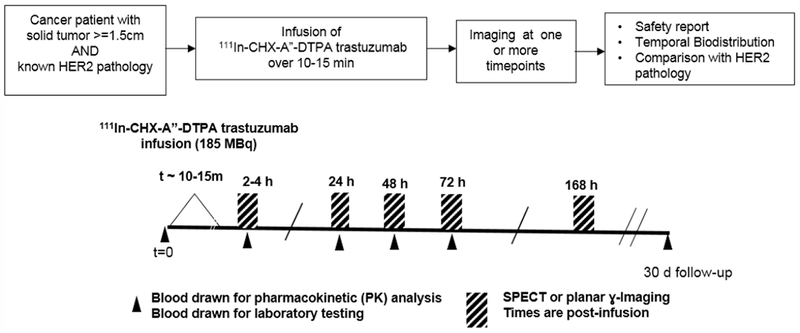
Protocol schematic

Whole-body planar imaging (Siemens ECAM, v6.5.9.12, 8cm/min, matrix: 512 × 1024 × 16, MCA IN-^111^In, 2-windows/l-channel) and/or regional SPECT (Siemens ECAM, v6.5.9.12, matrix:128 × 128,16-bit, 2-energy windows, filtered back-projection (FBP) reconstruction) or SPECT/CT (Phillips Healthcare Precedence 16P, vl.0, matrix: 128 × 128,16-bit, 2-energy windows, iterative reconstruction) was performed. All imaging for a given patient was performed on the same camera with prescribed imaging times of 2-4h, 24h, 48h, 72h and 168h. (Figure 1)

Visual interpretation was performed by at least 2 experienced Nuclear Medicine readers, blinded to HER2 status but aware of the location of the radiographic “target lesion” defined prior to patient enrollment. The imaging target was not always the lesion from which the pathologic HER2 determination was made. A patient was considered HER2(+) on imaging by visual analysis if any focal ^111^In -CHX-A”-DTPA trastuzumab uptake greater than background corresponding to a lesion on conventional imaging was identified. A patient was considered HER2(−) if no non-physiologic focal uptake was found at any of the imaging timepoints.

Up to 3 potential lesions were identified for each patient and regions of interest (ROIs) were created for each patient using an 80% “maximum pixel thresholding” tool (MIM 6.71). The resultant ROI included only the activity values of pixels within top 20% of values within the user-determined area. The average of the activity of this ROI was used to represent the maximum activity, limiting susceptibility to noise Normal organ uptake was defined by a l-2cm diameter ROI in a homogeneous region of the spleen, liver, lungs, brain, blood pool (BP) and soft tissues (ST). Additional ROIs were also drawn to contain the total activity within the liver, entire gastrointestinal (GI) track, entire small intestine (SI) and whole-body (WB). For planar images the geometric mean of the anterior and posterior projection values was used unless the focus was known to reside more anteriorly or posteriorly, in which case the mean ROI value from the respective image was used. As a 3D modality with limited field of view (FOV), SPECT imaging of the torso only was performed (excluding the brain and lower extremities). A surrogate WB ROI was defined only if the entire torso was imaged. Trastuzumab does not cross an intact blood-brain barrier, no specific activity was present in the brain on planar imaging and none of the target lesions were in the brain thus the torso ROI is a reasonable estimate of whole-body uptake. The maximum, minimum, median, mean, standard deviation(SD) and total counts were recorded for all ROIs at each timepoint. All imaging data were decay corrected to the time of injection, thus representing the physiologic biodistribution.

Planar and SPECT ɣ-photon imaging both pose challenges for obtaining semi-quantitative parameters, in part due to limitations correcting for soft tissue attenuation, high noise, low count rates, breathing artifacts, partial volume, spillover effects etc. To limit these effects and allow for comparison across patients and imaging sessions, representative organ ROI values were normalized to the ST ROI values. The ST uptake was chosen as it is least likely to have specific uptake and is less sensitive to differences in blood activity (which is dependent on the blood levels of trastuzumab (both cold and radiolabeled)) yet retains dependencies on the patient body habitus, injected dose, time and imaging platform. For focal lesions, a Tumor to Background ratio (T:B) was used (due to differences in background tissue distributions) and a ratio cut-off ≥1.5 was used to designate a lesion HER2(+). Time activity curves (TACs) were created for each ROI in patients who underwent imaging at multiple time points.

### Statistics

Due to the small patient population no statistical comparisons among patient were performed. Curve fitting using non-linear regression based on a single exponential, linear regression, and trapezoidal-based estimates for area under the curve (AUC). For nonlinear regression, the t_1/2_, R^2^ and 95% Confidence Intervals (CI) were reported and for linear regressions, the x-intercept, R^2^, and 95% CI were reported. These statistics and plot creation were performed using Prism GraphPad 7.01.

## Results

A total of 13 patients were enrolled; however, only 11 were imaged. One patient withdrew prior to receiving ^111^In-CHX-A”-DTPA trastuzumab and another patient received the dose but died from her disease prior to imaging (cause of death determined to be unrelated to the investigational imaging agent). Tissue samples used for the pathologic HER2 designation were obtained <3 months (2 patients), < 6-months (4 patients), <12 months (8 patients) and >12 months (3 patients) prior to imaging. Imaging of the biopsied lesion occurred in only 4 patients whose tissue samples were obtained 0.7, 3, 10, and 22 months prior to imaging. While not statistically evaluated, (Table 1) EBD levels did not appear to correlate with pathology or imaging positivity. As the units of our results were not in the range of those reported prior publications (pg/ml vs ng/ml or μg/ml) [30–32] we performed further validation testing on HER2(+) patients as described in the Supplementary Data (S1). The differences in the scale is likely due to the detection antibodies used.

The mean radiochemical purity of >95% was achieved for all clinical doses (99.25%, SD 0.69%). (Table 2) The sterility testing showed no growth after 14 days and LAL testing was negative in all patient doses. The safety laboratory results were within values expected for the current course of therapy. The 30-day follow-up serum/plasma samples showed no evidence of specific antibody formation. Analysis of patient blood samples showed greater than 90% of activity in the plasma after separation from the cellular components, consistent with previous reports [33].

^111^In-CHX-A”-DTPA trastuzumab mean administered dose was 174.5 [123.4-194.2, SD 21.3]MBq, mean mass 133 [70-169, SD 37.5] μg. (Table 2) One patient experienced a Grade 1 taste disturbance during the infusion that resolved spontaneously. Whole-body planar imaging was performed at multiple time points on 6 patients, torso SPECT/CT on 5 patients and 2 patients underwent both planar and SPECT imaging. Not all patients were able to be imaged at all scheduled timepoints and 4 patients only underwent a single SPECT/CT image. All imaging for a given patient was performed on the same camera.

Data from multiple planar imaging sessions was available for 6 patients. (Figure 2A) shows an example of a HER2 True Positive patient’s imaging series. (Figure 2B) displays a graph of the T:B parameter for each individual patient as well as the average of all patients designated HER2(+) by imaging. Visual and image-based analyses were concordant in all patients. All tissue HER2(+) patients had T:B >1.5 beginning with the initial 2–4h imaging session. While there was mild variability of T:B values with time, HER2(+) lesions remained >1.5 at all timepoints. Visual analysis definitively identified the HER2(+) lesions by 48-72h at the latest. Image-based and tissue-based HER2 designation were congruent in all but 1 patient. (Table 1) This patient had metastatic NSCLC which was HER2(−) by pathologic criteria (based on a pericardial effusion sample: HER2 2+, FISH 1.2); however, focal uptake in a right lung lesion was apparent, best visualized at the 72h time point with a positive T:B value of 6.7, remaining >1.5 across all timepoints. The second HER2(−) patient in this subset had no visible focal uptake and an ROI of the target lesion location maintained a T:B <1.5 (True Negative). The third HER2(−) patient underwent a single timepoint 72h post injection SPECT/CT with a T:B of 0.87 (True-Negative). (Figure 3A) shows an image from the imaging/pathology False Positive patient and (Figure 3B) an example of an imaging/pathology negative patient (True Negative).

At the initial imaging time-point prominent activity was noted in the liver, spleen, BP and BM. The average BP:ST decreased with time, fitting to a single-phase decay (t_1/2_ =34.2h, 95%CI 25.3 to 46.3h; R^2^ = 0.96). (Figure 4A) The average spleen: ST TAC was mildly variable, with minimal decrease followed by an upward trend. The average TACs for the total counts normalized to the injected dose for the WB, liver and GI tract are depicted in Figure 4B. The average liver activity TAC normalized to injected dose (ID) peaked at the initial image (mean 3.3h) fitting to a single-phase decay using non-linear regression estimated t_1/2_=46.9h (95%CI 41.8 to 53h; R^2^=0.97). Evaluating the area under the curve (AUC) for the average liver fraction of WB activity (average for measurement for each patient at each timepoint) indicated a peak fraction of 0.47 at 3.3h with the AUC total representing 48.9% [95%CI 33.6 to 64.2%] of the WB AUC. The GI fraction peaked at 51.7h with a WB fraction of 0.39 and estimated GI contribution 51% [95%CI 44.1 to 58%] of the decay corrected total WB activity over time. (Figure 4C) Linear regression beginning at the peak activity (51.7hr) estimated a time to complete GI clearance of 201.2h (95%CI 188.5 to 216.9h; R^2^ =0.963). The average WB activity began to decrease following the initial timepoint. Linear regression fit estimated complete clearance by 256.5h, 95%CI 186.1 to 489.2h; R^2^=0.967. (Figure 4B) Across all timepoints, there was only mild uptake in the small bowel with the gall bladder distinctly visualized at 72h in two patients. The kidneys were visualized on the initial images but had minimal uptake over time. The pelvis had only mild uptake throughout (excluding the bowel).

## Discussion

HER2 over-expression is present in numerous malignancies [34] and may respond to HER2 directed therapies such as trastuzumab, lapatinib, pertuzumab, ado-trastuzumab emtansine and neratinib. As more therapeutics demonstrating effectiveness in HER2(+) patients emerge it becomes even more important to develop a global, non-invasive yet accurate means of identifying patients who may benefit. Despite the availability of consensus recommendations by ASCO and CAP, for determination of tissue HER2 expression levels in breast cancer (updated most recently in 2016)[35] and the availability of FDA-approved testing methods/kits [16], compliance has been questioned. A study comparing central versus local laboratories showed only a 72% concordance rate [18]. Inconsistency is inherent in the complex process involved with possible discrepancies arising from specimen quality [36], tumor heterogeneity [37], antibody selection [17], post-staining interval [38], and interpretative subjectivity [39].

Our data support the concept that whole body imaging could assist in the non-invasive assessment of HER2 expression across multiple lesions. Reports in the literature show high rates of discordance between HER2 expression in the primary tumor and metastatic foci [21], making an argument for whole-body evaluation in patients with metastatic disease. ^111^In-CHX-A”-DTPA trastuzumab demonstrated excellent imaging characteristics without the pre-administration of trastuzumab. The safety of this agent was excellent with no Grade 2 or greater adverse events. Visual analysis and semiquantitative analysis were concordant in all cases and comparison with pathological HER2 results on a patient basis was good (agreement in 10 of 11 patients). The only mismatch was a false positive, wherein the tissue used for HER2(−) designation was not obtained from the target lesion. A core needle biopsy of the right lung mass was performed but the sample proved inadequate for HER2 testing. Given the frequency of heterogenicity within and among tumors, it is quite possible that the metastatic lung focus exhibited HER2 over-expression despite the HER2(−) designation on the pericardial effusion.

Previous reports on HER2 imaging agents focused primarily on plasma pharmacokinetics and lesion uptake. With imaging, it is possible to track the biodistribution of ^111^In-CHX-A”-DTPA trastuzumab over time. Our BP clearance estimates (t_1/2_ of 1.4d) fall in line with previously published data demonstrating a plasma clearance 1.7d t_1/2_ at 10mg trastuzumab dose [13]. The literature also shows that the blood clearance is bi-phasic (initial fast α-phase followed by a slower β-phase clearance) with longer t_1/2_ values as administered dose increased. With a daily administration of 50mg, the t_1/2_ published was plasma 5.8d, and at low therapeutic dose (2mg/kg) following a loading dose the t_1/2_ had a mean value of 5.84d, range 1 to 32d [13]. As we did not pre-administer any trastuzumab and the mean administered radiolabeled dose was 133ug, our findings fall in line with the rapid α-phase. Only 1 patient on therapeutic levels of trastuzumab during our protocol underwent multiple time point imaging so subgroup analysis of BP values in patients on trastuzumab was not performed. Unfortunately, we did not measure trastuzumab blood levels so comparison of ^111^In-CHX-A”-DTPA trastuzumab distribution with trastuzumab blood concentrations were not possible.

Like many other antibody-based imaging agents, hepatobiliary excretion was prominent (~54% of the whole-body clearance) resulting in high liver and GI activity in the abdomen/pelvis; however, liver and GI activity distributions are temporally separated. (Figure 4) Dual time point imaging and appropriate image scaling may overcome the potential limitation of lesion classification in the liver, abdomen and pelvis. The liver clearance is rapid with t_1/2_ estimated ~2d (although the 95%CI extended to ~6d). The projected GI clearance was more prolonged, estimated at 8.3d. Our data suggest a significant alternate elimination route as ~50% of the whole-body activity is left unaccounted. Assuming the whole-body elimination is linear, our data predicts ~11d to achieve complete clearance (x-intercept of the linear regression of the WB TAC), although there is significant variability in this estimate as the high end of 95%CI is 25d. Our estimates are limited by small number of subjects and timepoints: imaging to only 7d. A linear clearance assumption for the GI and WB activities may not be appropriate. Both ADCP and ADCC are known mechanisms of action of trastuzumab and the slow trend toward increasing splenic uptake with time noted would be consistent with antibody opsonization. With low BP at the later timepoints, prolonged GI uptake via opsonization and/or phagocytosis may contribute to a more protracted elimination via a non-linear excretory pathway.

While several other promising agents are under development using antibody fragments, nanobodies, and other smaller entities to improve clearance, we limited our comparisons to radiolabeled trastuzumab imaging agents with published reports of use in humans. (Table 3) Prior reports of imaging using SPECT and PET radionuclides chelated to the whole trastuzumab antibody have been performed in HER2(+) breast cancer patients scheduled to receive trastuzumab therapy, typically incorporating a loading dose of “cold” (non-radiolabeled) trastuzumab. Pre-dosing with cold trastuzumab is intended to decrease the visualization of the initial fast (α) blood clearance component by the liver and allow for imaging during the longer β component of the biphasic blood clearance of trastuzumab [13].

Both Perik *et al.* [22] and Gaykema *et al.* [24] described imaging studies using ^111^In-DTPA trastuzumab in known HER(+) breast cancer patients administering cold trastuzumab prior to imaging agent infusion. While the biodistribution was not discussed by Perik *et al.* [22] who focused on cardiac uptake, an image in the publication showed high liver activity with visual identification of a liver mass displaying significantly higher radionuclide retention, demonstrating that HER(+) liver lesions can be visualized despite high background activity (and trastuzumab pre-treatment). Gaykema *et al.* imaged both pre- and post-trastuzumab therapy, reporting measurable changes following therapy (<20%) and overall improved tumor visibility with longer post-infusion imaging times. They observed persistence of high blood pool through 72h on the post-therapy patients and recommended imaging be performed after 4 days. Wong *et al.* [23] described ^111^In-MxDTPA trastuzumab imaging in a similar patient population, also pre-treated with cold trastuzumab. Imaging over time they estimated a plasma t_1/2β_ of 225hr which is much longer than our estimate; however, as we did not give a pre-dose, our data is expected to represent the faster t_1/2α_ component. With prior documentation that the biodistribution for ^111^In-MxDTPA- and ^90^Y-MxDTPA-trastuzumab were similar, if not identical, they reported the organ absorbed doses based on the therapeutic radionuclide ^90^Y (β^−^ emitter). With either ^111^In-trastuzumab agent, both the liver and myocardium were among the 3 organs receiving the highest doses.

Dijkers *et al.* confirmed the blood clearance dependence on trastuzumab blood concentrations using ^89^Zr-N-SucDf-trastuzumab (^89^Zr t_1/2_=78.4h) in mice. Their data revealed that tumor uptake was not dose dependent, but the normal organ distribution/clearance was [40]. In a separate study [41], patients underwent PET imaging with only 37 MBq of ^89^Zr-N-SucDf-trastuzumab IV with good imaging quality. The modality’s higher sensitivity and resolution compared with SPECT yielded higher quality quantitative images despite the low radioactivity of the administered dose. Based on initial data (n=2) they concluded a 10mg trastuzumab pre-dose was inadequate as the ensuing rapid α-phase blood clearance did not permit enough blood activity for tumor uptake (while no lesions were seen in one patient, multiple were visualized in the 2^nd^ patient). Both their animal study (above) and our results challenge that assumption. Based on clinical imaging, they concluded that imaging should be performed between 4–5 days after dosing. ^89^Zr-N-SucDf-trastuzumab’s bio distribution effectively decreased background and increased the tumor to background ratios while retaining enough radioactivity for PET imaging. While our data showed rapid blood clearance and initial high liver uptake, the liver clearance was rapid and followed by a prominent GI distribution. The consistent or mildly increasing T:B values permitted tumor visualization and measurement in all HER2(+) patients we imaged. Our data suggest that imaging >72hr post ^111^In-CHX-A”-DTPA trastuzumab administration may improve visualization due to the decreasing background. Although further study may prove addition of a cold trastuzumab pre-dose beneficial, it is apparent that delayed imaging is required regardless.

The stabilization or increase the T:B over time found in our data and the above reports is consistent with tumor retention of HER2 EBD-^111^In-trastuzumab or −^89^Zr-N-SucDf-trastuzumab complexes either intracellularly or on the tumor surface were independent of blood clearance. In vivo evidence suggests that HER2(+) cells traffic HER2(−) trastuzumab complexes differently than HER2(−) cells. While the later routes the complexes to the lysosomal degradation pathways following endocytosis, HER2(+) cells retain the EBD-trastuzumab complexes, permitting decreased degradation and recycles them to the surface [42]. In addition to the initial presence of more HER2 receptors (~10^6^ for a HER2 3+ cell), this retention and recycling increases the duration of trastuzumab’s residence in/on the cell [42], increasing signal.

O’Donoghue *et al.* reported on HER2(+) gastro-esophageal cancer patients imaged after administration of a trastuzumab pre-dose and ^89^Zr-N-SucDf-trastuzumab[43]. Their measured the median plasma and WB clearance rate predictions were higher than ours. Table 3 The slower BP clearance is expected due to higher doses of the antibody and may contribute to a prolonged WB clearance, but our estimates are based on a low number of patients with a non-quantitative imaging method (TACs were constructed from planar images) and their estimates are within the higher end of our 95%CI.

^64^Cu-DOTA trastuzumab PET/CT has also been studied in 6 breast cancer patients (3 primary and 3 metastatic HER2(+) disease) with at least 1 lesion visible by PET/CT on all patients imaged [44]. It is only other radiotracer study reported that uses the entire trastuzumab antibody imaged without the pre-administration of cold trastuzumab; however, 4 of 6 patients were on trastuzumab therapy. The reported temporal biodistribution of ^64^Cu-DOTA trastuzumab was quite different than for ^111^In-CHX-A”-DTPA trastuzumab, possibly due to the DOTA chelate. Liver and bladder activities were high, and the liver shows a slow increase with time. In part likely due to the shorter t_1/2_ of ^64^Cu (12.7h), 2 days was the recommended time to image for this agent. An additional published report of ^64^Cu-DOTA trastuzumab in breast cancer focused on metastatic brain lesions; however, it is difficult to separate non-specific accumulation from blood-brain barrier breakdown and specific binding in a short period of time [45]. The patient population appeared to overlap that of the prior report. Like ^89^Zr, with decay emissions of only 22.8% PET imageable β^+^, ℇ=0.22MeV (78.2% EC and 99% γ-emission ℇ=909keV via ^89m^Zr decay), ^64^Cu emits 18% PET imageable β^+^, ℇ=0.28 MeV (39% β ℇ=190keV and 76%EC ℇ=183-581keV), The shorter t_1/2_ of ^64^Cu raises questions about its suitability as a label for whole antibodies such as trastuzumab.

While availability and production costs of ^89^Zr and ^64^Cu may limit rapid clinical adoption, they are both safe and sensitive. ^111^In-CHX-A”-DTPA trastuzumab is a reasonable and economically feasible imaging agent which is also safe and sensitive (based on this small pilot study), albeit limited in resolution. The precursor is exceptionally stable and can be stored for long periods. Further studies evaluating the relationship between plasma trastuzumab concentration and bio distribution as well as image guided biopsies are needed to validate ^111^In-CHX-A”-DTPA trastuzumab as an imaging biomarker. Effects of higher trastuzumab concentrations and/or combination with other HER2 targeting agents on bio distribution may yield much useful information. Such issues are also critical for planning targeted therapeutic radiopharmaceutical dosimetry.

## Conclusion

^111^In-CHX-A”-DTPA trastuzumab can be safely administered and imaged in patients without the addition of non-labeled trastuzumab. Visual analysis was concordant with pathological HER2 designation in 10 of 11 patients. Preliminary data suggest the objective measure, T:B ratio ≥ 1.5 appears to be a reasonable cut-off for defining imaging HER2 positivity. The relatively low cost, availability, ease of synthesis, high chemical purity, specific activity combined with the imaging results of this first-in-human imaging study of ^111^In-CHX-A”-DTPA trastuzumab whole-body imaging HER2 expression lays the groundwork for future larger clinical validation studies. ^111^In-CHX-A”-DTPA trastuzumab may be a clinically useful diagnostic and/or therapeutic radiopharmaceutical for HER(+) tumors.

## Supplementary Material

S1

## Figures and Tables

**Figure 2A.**
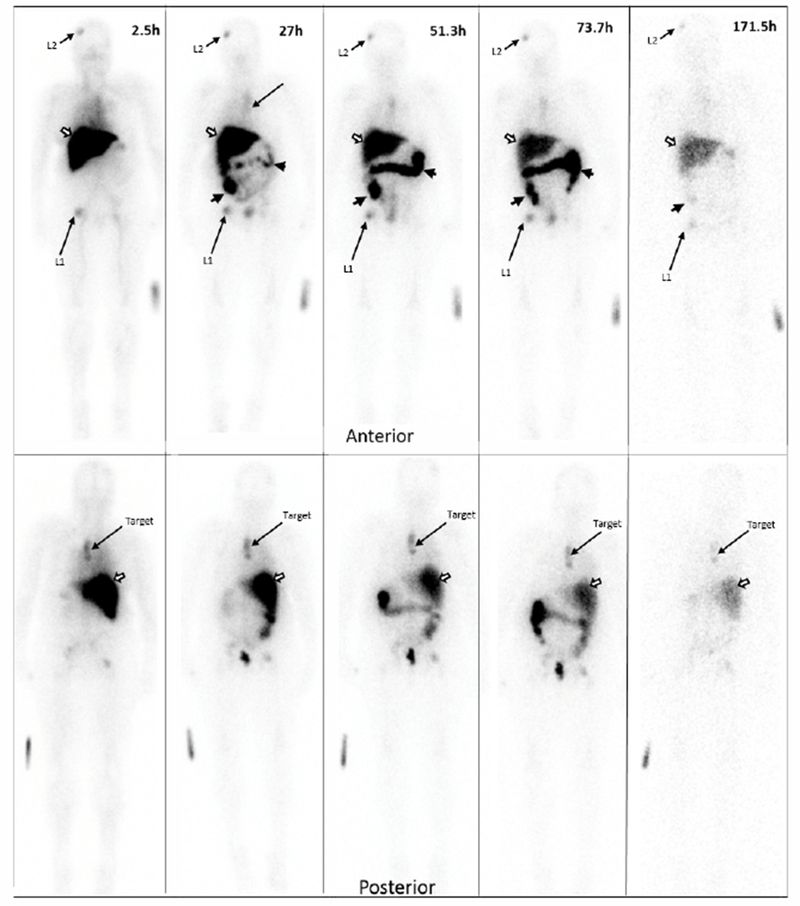
Planar imaging of a HER2 True Positive patient over time. Serial Anterior (top row) and Posterior (bottom row) Planar imaging of a breast cancer patient following the i.v. administration of 186.8 MBq (69.7μg) of ^111^In-CHX-A”-DTPA trastuzumab (23.7-minute wholebody acquisition, 1.3mm/s, 1.85m). The HER2(+) left paraspinal mass (“target” on the bottom row) shows focal ^111^In-CHX-A”-trastuzumab uptake. Other prominent foci in the right frontal bone (L1) and right hip (L2) are consistent with patient’s known metastatic bone disease. The remaining distribution is physiologic, seen throughout all patients: accumulation of tracer in the liver (white arrow) and large bowel (black arrow heads) and no significant brain or cardiac uptake. Times indicate time post-injection. Images are scaled to represent biological changes only. Radioactive decay is NOT depicted. Degradation of imaging quality at the 171.5h time point is due to low counts.

**Figure 2B.**
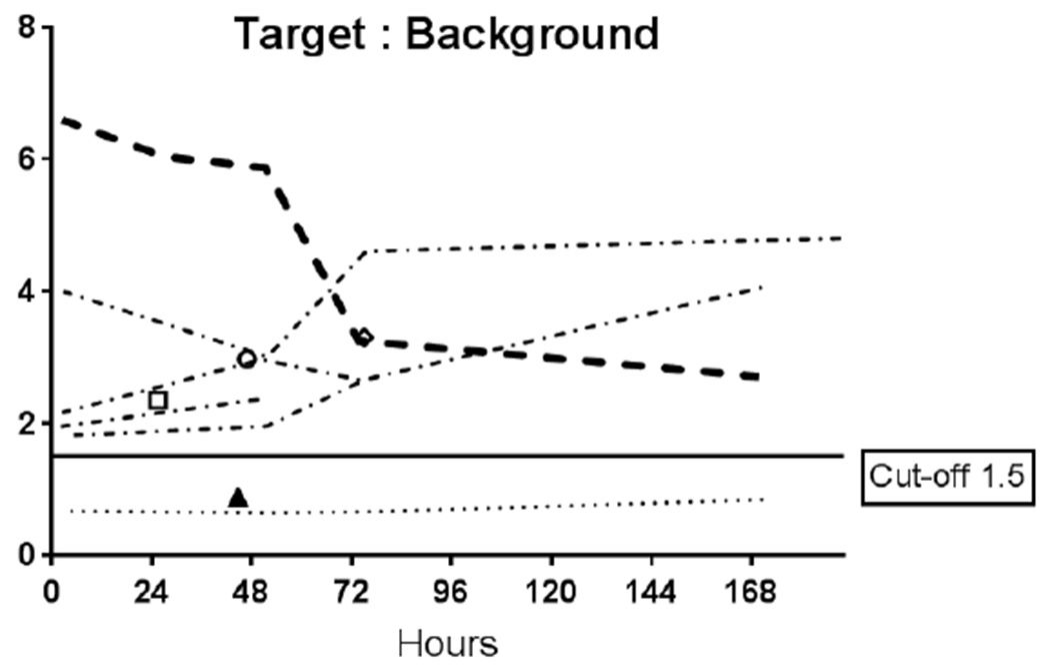
Target tumor to background (T:B) Time Activity Curves (TACs) Target tumor to background (T:B) Time Activity Curves (TACs) for each of the 6 patients who underwent serial planar imaging. The thick dashed line is the TAC for patient 001 (2a). Symbols in the plot represent T:B of patients who were only imaged at a single time point. Only 2 of 3 the HER2(−) patients had L:B <1.5. All the HER2(+) patients’ ratios were above the 1.5 cut-off. B

**Figure 3A.**
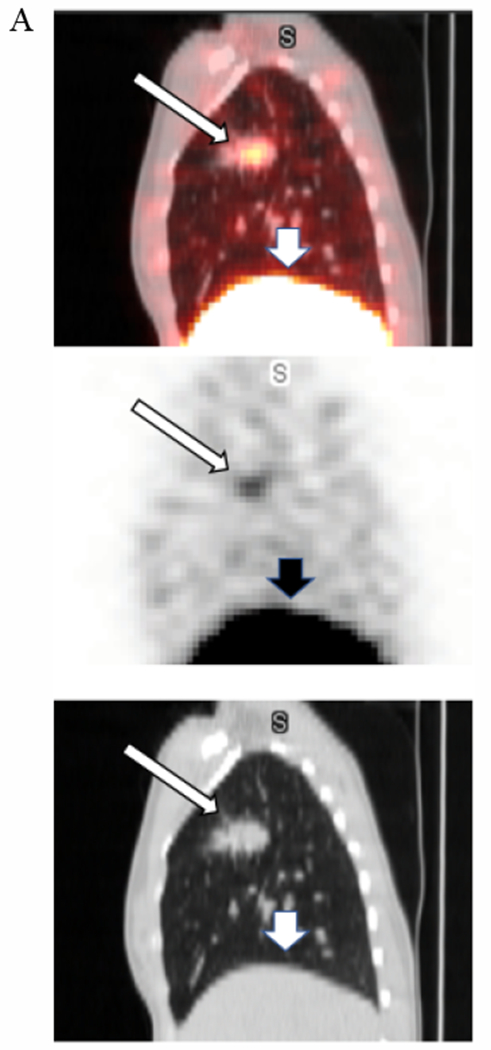
False positive. A 35-year-old female with NSCLC enrolled onto our study as HER2(−) based on pericardial effusion biopsy (HER2 2+; FISH ratio 1.22). The right upper lung lesion (the Target Lesion) is easily visualized (narrow arrows) on these fused SPECT/CT, SPECT, and low attenuation CT sagittal projections from the 71h post iv infusion of 128.4MBq[160.4ug] ^111^In-CHX-A”-DTPA trastuzumab imaging session. Tumor to Background Ratio is 6.7. The wider arrows show the liver.

**Figure 3B.**
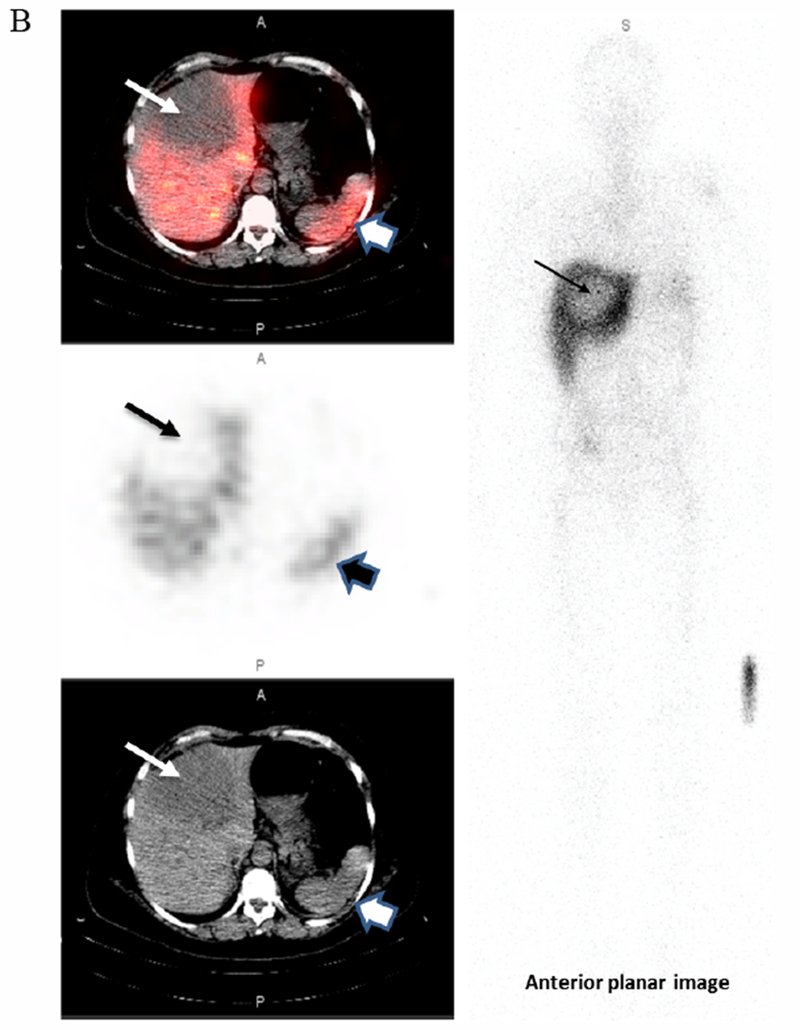
True negative. This 56-year-old female with metastatic breast cancer presented with a large hypodense liver lesion (narrow arrow on left) seen best on the low dose CT scan (bottom image). Biopsy showed no evidence of HER2 overexpression (HER2 1+; FISH 1.6). SPECT images performed at 173h post-infusion of 189.8MBq[136.5μg]. ^111^In-CHX-A”-DTPA trastuzumab shows no significance uptake compared to liver background (top SPECT and middle fused SPECT/CT image). Lesion to Background ratio is 0.85. The wider arrow on the right indicates the spleen. Some mis-registration is present likely due in part to respiratory motion

**Figure 4A.**
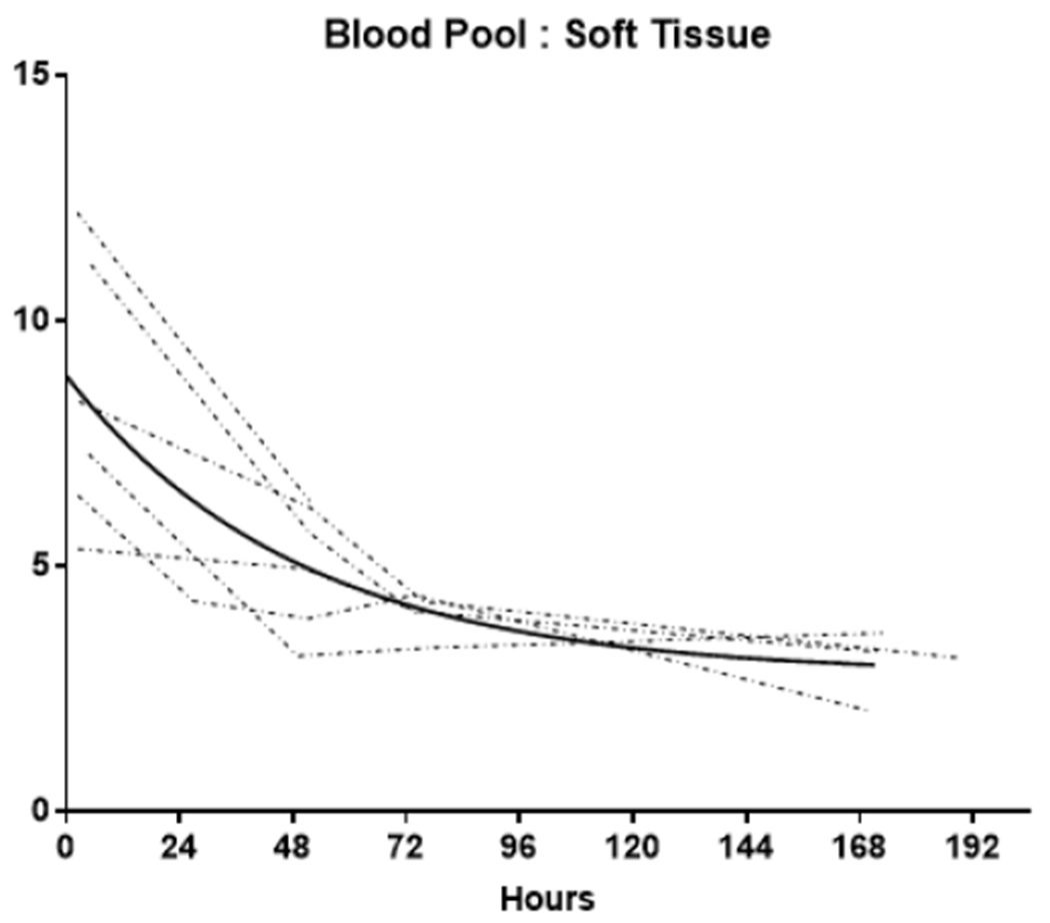
Blood pool: Soft tissue ratio (BP:ST) time activity curves (TACs) Individual TACs of the BP:ST ratios for each patient imaged at multiple time points. The solid line represents the non-linear regression single exponetial fit (GraphPad Prism v7.01). Fit parameters: R^2^= 0.96; t_1/2_ = 34.2h [95% Confidence Interval: 25.3 to 46.3h]

**Figure 4B.**
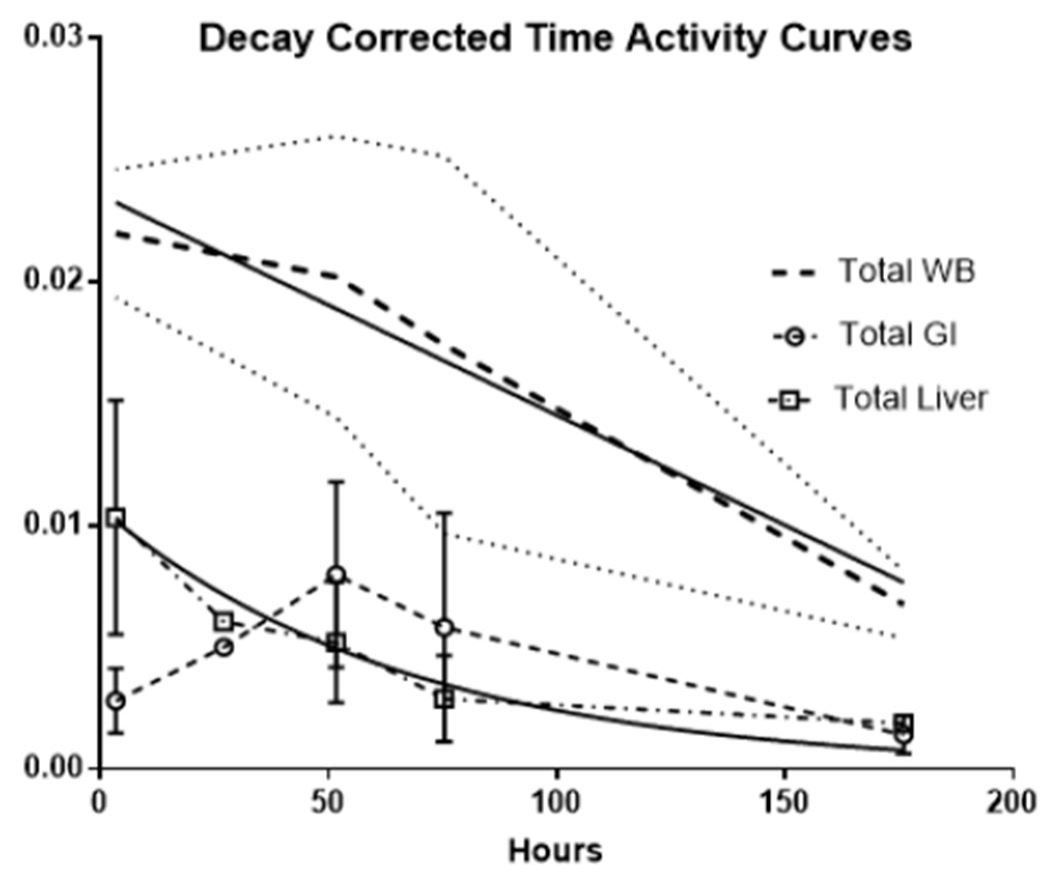
Decay corrected total activity curves The decay corrected total whole-body (WB) activity over time is depicted by the heavy dashed lines, the thin dotted line on either side are the standard deviation (among patients). The decay corrected total gastrointestinal and liver time-activity curves (TACs) are indicated by the dashed open circles and the dash-dot open squares respectively with the vertical line representing the standard deviation among individual patients**’** data. The solid lines are the linear regression fits for the WB and GI and the non-linear regression single decay fit for the liver

**Figure 4C.**
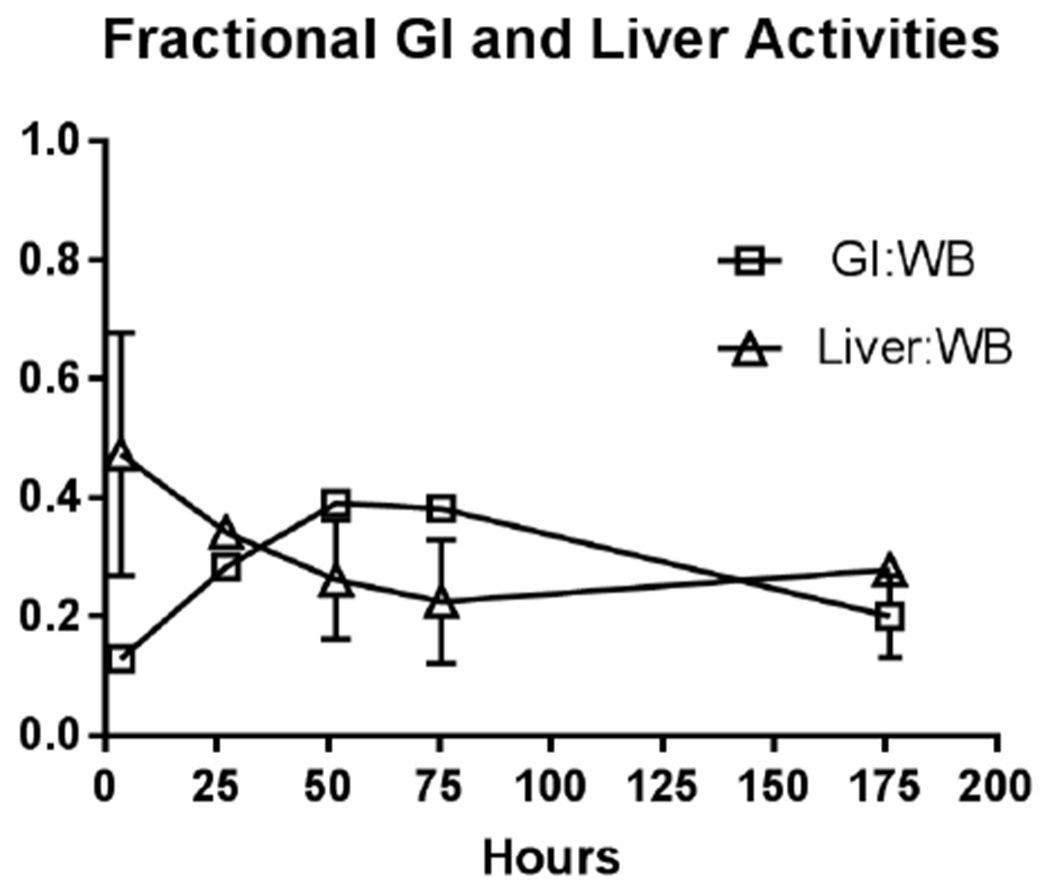
Fractional contribution of the liver the GI tract to the decay corrected whole body counts Average of individual patients**’** ratio of decay corrected total GI track and total liver activities to decay corrected WB activity respectively. Vertical bars represent standard deviation (across patients). These are NOT plots of the GI and Liver total activities normalized to the injected dose (found in Figure 4B), but that of fractional contribution of the GI and Liver to Wholebody activity respectively. Comparison of area under the curve (AUC) values show the liver and the GI contribute 50% of the wholebody activity over the entire time imaged. Thin lines indicate the standard deviation of the WB TAC

**Table 1.** Patient and tumor characteristics. Clinical, pathology, and imaging data for all images patients is provided. All patients had received prior chemotherapy. Three patients were on trastuzumab during the study and 8 patients received it previously. There were 8 HER2(+) and 3 HER2(−) patients by pathology. One non-small cell lung cancer patient (011) had a well-defined right lung lesion on CT and ^111^In -CHX-A”-DTPA trastuzumab imaging but was HER2(−) by pathology based on a pericardial fluid sample, thus designated an imaging False Positive. Tissue from the right lung mass was submitted for review by was inadequate for HER2 analysis. Six of the tumors biopsied were the primary tumors, surgically excised prior to enrollment. Only 4 patients’ tissue samples were of the tumor imaged. Levels of “shed” HER2 Extracellular Binding Domain (EBD) in the plasma were measured; however, no correlation with either pathology or imaging HER2 findings was evident.

				Current Chemotherapy							Pathology HER2	
001	52	F	Breast	Femara	Paraspinal Mass	6.1	53.3[0.4]	Left breast mass	3+	NA	+	+
002	58	F	Lung	Lapatinib + capecitabine	Left Upper Lung	5.6	26.9[6.6]	Left breast mass	3+	NA	+	+
003	67	M	Lung	Erlotinib	Left Pleural Effusion	3.5	BLQ^a^	Pleural fluid	2+	2.8	+	+
004	53	M	Breast	Tarceva	Posterior Left Lower Lung	4.4	1215.1[58.1]	Left lung mass	3+	4.3	+	+
006	68	F	Breast	Gemcitabine, D + T	Left Lung Mass	4.0	742.3[22.8]	Left breast mass	3+	4.7	+	+
007	56	F	Breast	none-in washout period	Liver	3.0	BLQ	Liver mass	1/2+	1.6	−	−
009	48	F	Breast	T + navelbine	Sternum	1.5	38.9[0.58]	Sternal (mass FNA)	3+	2.5	+	+
010	62	F	Breast	s/p XRT, capecitabine	Right para-iliac node	1.8	BLQ	Left breast mass	1 +	NA	−	−
***011***	**35**	**F**	**Lung**	**AdHER2 Vaccine**^b^	***Right Upper Lung***	**2.0**	**BLQ**	***Pericardial effusion***	***2***+	***1.22***	−	+
012	43	M	Bladder	AdHER2 Vaccine^b^	LLL/B ladder	3.7	18.1[0.60]	Bladder	3+	0.85	+	+
013	61	F	Breast	D + T + P	Right Lung	1.6	BLQ	Left Breast	3+	NA	+	+

a Below Level of Quantification <8.2 pg/ml

b Patients who received AdHER2 vaccine were also enrolled in a separate clinical trial: A Phase I Study of an Adenoviral Transduced Autologous Dendritic Cell Vaccine Expressing Human HER2/Neu ECTM in Adults with Tumors With 1–3+ HER2/Neu Expression. ClinicalTrials.gov ID: NCT01730118

(D = docetaxel T = trastuzumab P = pertuzumab).

**Table 2.** 111In-CHX-A”-DTPA trastuzumab administered doses

^111^In-CHX-A”-DTPA trastuzumab					
	Radiochemical Purity (%)		Administered Dose Parameters		
Patient ID	ITLC (Instant Thin Layer Chromatography)	Paper Chromatography	Activity (MBq)	mass (μg)	Specific Activity (MBq/μg)
001	98.8	98.05	186.85	69.75	2.68
002	99.4	99.70	142.82	159.96	0.89
003	99.9	99.09	185	70.13	2.64
004	99.1	99.36	161.69	161.41	1.00
006	99.9	98.09	188.7	126.48	1.49
007	100	100.00	189.81	136.50	1.39
008^#^	100	98.50	179.08	144.20	1.24
009	99.4	99.16	186.48	168.58	1.11
010	100	99.50	184.63	88.65	2.08
011	99.4	98.13	128.39	160.38	0.80
012	100	100.00	173.53	157.68	1.10
013	97.9	98.70	193.88	104.54	1.85
**Average[SD]**	**99.48 [0.64]**	**99.02 [0.72]**	**175.1 [20.5] MBq**	**129.0 [36.8]**	**1.52 [0.6]**

Twelve patients received 1 111In-CHX-A”-DTPA trastuzumab infusions; however, one patient (008) died of her disease prior to imaging. All clinical doses produced met Quality Control Acceptance criteria including radiochemical purity >95% and chemical mass <200μg

**Table 3.** Comparison of clinical radiolabeled trastuzumab studies

Author	Agent	t_1/2_(h)	Tumor Type	n	HER2 Status	Trastuzumab pre-dose	Administered Dose	Lesion visibility	Organs receiving highest dose
Perik et al.[1]	^111^In-DTPA trastuzumab	67.9	Breast	17	All 3+	4mg/kg	100-150 MBq/5mg	At least 1 in 14/15 patients New lesions in 13/15	na
Gaykema et al. [2]^1^	^111^In-DTPA trastuzumab	67.9	Breast	12	+	4mg/kg + 2mg/kg weekly	100-150 MBq/5mg	25	liver, spleen, Myocardium
Wong et al [3]	^111^In-MxDTPA trastuzumab	67.9	Breast	8	≥3+	4-8 mg/kg	185 MBq/10mg	At least 1 in 3/7 patients	myocardium, liver, kidneys^2^
Dijkers et al[4]	^89^Zr-N-SucDf-trastuzumab	78.4	Breast	14	+	10[n=2], 50[n=5] OR 10mg[n=7] if on trastuzumab	38.4[1.6] MBq/1.5mg	All lesions in 6/12 patients	na
O’Donoghue et al[5]	^89^Zr-N-SucDf-trastuzumab	78.4	Gastroesophageal	10	3+(n=8) 2+FISH+(n=2)	50 mg	184(182 to 189) MBq /3mg	At least 1 in 8/10 patients	Liver, myocardium, kidneys
Tamur et al.[6]	^64^Cu-DOTA trastuzumab	12.7	Breast	6	3+(n=5) 2+FISH+(n=1)	86.2±6.3 μg	126±8MBq	At least 1 in 6/6 patients 9/11 lesions	myocardium, liver, spleen
Data from this manuscript	^111^In-CHX-A”-DTPA trastuzumab	67.9	Breast (n=8), NSCLC (n=2), Bladder (n=1)	11	3+(n=7) 2+FISH+(n=1) HER2(−)(n=3)	129.02[36.8] μg	175.7[20.3] MBq	10/11 Concordant with pathology	liver, bowel, myocardium

na = not available;

1 same patient population as above;

2 dose calculations using ^90^Y

Current publications of radiolabeled trastuzumab clinical imaging are limited by small numbers of patients and variable patient preparation and imaging protocols. Our is the only study that included HER2(−) patients and 1 of 2 in which additional non-radiolabeled trastuzumab was NOT given. Despite the numerous inconsistencies, HER2 imaging with radiolabeled trastuzumab is safe and feasible with PET or SPECT; however further validation studies with larger patient populations are needed
