# Supplementary material for: First-in-human phase 0 study of 111In-CHX-A”-DTPA trastuzumab for HER2 tumor imaging
Source: J Transl Sci. Author manuscript; Available in PMC 2019 Apr 1. (PMC6425962; doi:10.15761/JTS.1000269)
Supplement: S1 [file NIHMS989713-supplement-S1.pdf]

S1

The units for concentrations of shed HER2 extracellular domain (ECD) we reported (pg/ml) are lower than most reported in manuscripts measuring shed HER2. The 2000 the 1st FDA-approved standardized and quantitative automated ELISA, the Bayer Immuno 1, suggested a cut-off 15 ng/ml, greater than which is considered positive for shed HER2 ECD[1]. By that criteria none of our patients had detectable levels of shed HER2 ECD. Since then other validated methods for shed HER2 ECD have been described[2].

We utilized a HER2 ELISA kit from Millipore Sigma (St. Louis, MO, USA) that uses a monoclonal mouse capture antibody derived from a mouse myeloma cell line NS0-derived recombinant human ErbB2 (aa23-652) with a goat polyclonal detection antibody derived from the same immunogen. We used fresh frozen human plasma and serum derived from each of three patients with HER2(+) breast cancer participating on a HER2 dendritic cell vaccine trial (NCT01730118) to validate our results. Patients 011 and 012 in our imaging study were also participating in NCT01730118, although not included in this validation testing. Our assay (lower limits of detection is 8.2 pg/ml) detected the following plasma and serum concentrations, respectively: (1050pg/mL, 1651pg/mL, 1619pg/mL) and (179pg/mL, 135pg/mL, and 709pg/ml). Such concentrations approximated the average plasma (~1600pg/mL) and serum (~256pg/mL) concentrations obtained by Sigma, and the company confirmed our assay performed well (personal correspondence between Dr. TM Sissung and Millipore Sigma Technical Services on 3/16, 2018). While the results from the present assay are different from previously published concentrations, this observation is most-likely caused by differences between the antibodies used in the Sigma ELISA and the commonly used Wilex assay from Martell Diagnostic Laboratories (Roseville, MN, USA; formerly Wilex Inc, Cambridge, MA, USA) [3].

1. Cook GB, Neaman IE, Goldblatt JL, Cambetas DR, Hussain M, Luftner D et al. Clinical utility of serum HER-2/neu testing on the Bayer Immuno 1 automated system in breast cancer. Anticancer research. 2001;21(2b):1465-70.
2. Carney WP, Neumann R, Lipton A, Leitzel K, Ali S, Price CP. Potential clinical utility of serum HER-2/neu oncoprotein concentrations in patients with breast cancer. Clinical chemistry. 2003;49(10):1579-98.
3. Banys-Paluchowski M, Witzel I, Riethdorf S, Rack B, Janni W, Fasching PA et al. Evaluation of serum epidermal growth factor receptor (EGFR) in correlation to circulating tumor cells in patients with metastatic breast cancer. Scientific reports. 2017;7(1):17307. doi:10.1038/s41598-017-17514-8.
